# Erosion of Digital Professionalism During Medical Students’ Core Clinical Clerkships

**DOI:** 10.2196/mededu.6879

**Published:** 2017-05-03

**Authors:** Arash Mostaghimi, Aleksandra E Olszewski, Sigall K Bell, David H Roberts, Bradley H Crotty

**Affiliations:** ^1^ Department of Dermatology Brigham & Women’s Hospital Boston, MA United States; ^2^ Harvard Medical School Boston, MA United States; ^3^ Department of Pediatrics Seattle Children’s Hospital University of Washington Seattle, WA United States; ^4^ Division of General Medicine Beth Israel Deaconess Medical Center Boston, MA United States; ^5^ Center for Patient Care and Outcomes Research Health Systems Research Unit, Division of General Internal Medicine Froedtert & Medical College of Wisconsin Milwaukee, WI United States

**Keywords:** professionalism, health information systems, undergraduate medical education, social media, medical informatics

## Abstract

**Background:**

The increased use of social media, cloud computing, and mobile devices has led to the emergence of guidelines and novel teaching efforts to guide students toward the appropriate use of technology. Despite this, violations of professional conduct are common.

**Objective:**

We sought to explore professional behaviors specific to appropriate use of technology by looking at changes in third-year medical students’ attitudes and behaviors at the beginning and conclusion of their clinical clerkships.

**Methods:**

After formal teaching about digital professionalism, we administered a survey to medical students that described 35 technology-related behaviors and queried students about professionalism of the behavior (on a 5-point Likert scale), observation of others engaging in the behavior (yes or no), as well as personal participation in the behavior (yes or no). Students were resurveyed at the end of the academic year.

**Results:**

Over the year, perceptions of what is considered acceptable behavior regarding privacy, data security, communications, and social media boundaries changed, despite formal teaching sessions to reinforce professional behavior. Furthermore, medical students who observed unprofessional behaviors were more likely to participate in such behaviors.

**Conclusions:**

Although technology is a useful tool to enhance teaching and learning, our results reflect an erosion of professionalism related to information security that occurred despite medical school and hospital-based teaching sessions to promote digital professionalism. True alteration of trainee behavior will require a cultural shift that includes continual education, better role models, and frequent reminders for faculty, house staff, students, and staff.

## Introduction

The increasing use of social media, cloud computing, and mobile devices challenges medical schools and teaching hospitals to guide students toward the appropriate use of technology [1]. Medical school curricula addressing professionalism increasingly include a component on “digital professionalism,” discussing social media, physician identity, privacy, and protection of electronic protected health information (ePHI) [2-5].

Despite teaching efforts and the emergence of guidelines [6-9], violations of professional conduct in the digital realm are common [10]. Sixty percent of US medical schools report incidents of medical students posting unprofessional content online, including violations of patient confidentiality at 13% of schools [11]. Breaches in privacy can lead to severe legal consequences resulting in student suspensions and institutional fines. Data suggest that rising third-year students may not appreciate security risks stemming from use of mobile devices, placing patient data, the student, the medical school, and the hospital at risk [12].

In addition to formal instruction, studies show that components of the “hidden curriculum [13],” including informal interactions and observations of others, exert a profound effect on the unprofessional behaviors of medical students [14-16]. This influence likely extends to behaviors related to information security and patient privacy, although these components of professionalism have not been studied [17-19]. This study aimed to explore the influences of both formal and informal education during clinical clerkships on medical students’ professional behaviors specific to appropriate use of technology by examining changes in medical students’ attitudes and behaviors at the beginning and conclusion of their core clinical clerkships.

## Methods

### Study Design, Participants, and Setting

To assess the changes in medical student attitudes and behaviors about digital professionalism, we modeled the approach of Reddy et al [20], creating a survey listing behaviors and asking students to report whether they observed or participated in each behavior and to rate the behavior as either professional or unprofessional. We administered an anonymous survey to medical students at a large teaching hospital at the beginning and end of their third-year core clinical clerkships. We invited all students who were doing their hospital-based clinical clerkship year at our hospital during the academic year 2012-2013 to participate in the cohort. Participation was voluntary. The Institutional Review Board at the Beth Israel Deaconess Medical Center approved the study as exempt.

#### Survey Development

Our survey (Multimedia Appendix 1) consisted of 35 technology-related behaviors related to clinical clerkships. We chose the behaviors to represent domains of privacy, information security, communications, and social media, boundaries, and online tone. These behaviors captured elements of digital identity and perceptions of technology usage in professional settings as well. We chose these behaviors based on the collective experience of the study team in clinical informatics (BC, AM), medical ethics (SB), and clerkship education and leadership (DR). Not all behaviors were incontrovertibly unprofessional, and several were intentionally “gray areas.” We asked three questions per behavior. We asked students to report yes or no whether they (1) observed and (2) participated in each behavior. Then, students were asked to (3) rate each behavior on a Likert scale from 1 “Very Unprofessional” to 5 “Very Professional.”

#### Educational Sessions

Immediately before their initial clinical clerkship, all students received two 45-min educational sessions on digital professionalism (Multimedia Appendix 2). The first occurred for all rising clinical clerkship year students as part of a central orientation, and the second occurred the following day as part of hospital-specific orientation activities for the smaller cohort of students doing their principal clinical experience at our hospital. Two faculty members (AM, BC) developed and led both sessions to provide students with information and education regarding workplace professionalism related to technology. Sessions included background about relevant issues, best practices for information security, and case-based discussions of digital professionalism with an emphasis on professional behavior. The first session introduced three cases to illustrate concepts of digital identity, information security, and perceptions of technology use. We developed cases to illustrate basic principles rather than provide prescriptive instructions, given that it would not be possible to cover all scenarios that students would be likely to encounter. The second session, conducted at the hospital, allowed for interaction and question and answer sessions, as well as discussion of policies and procedures for information security.

#### Survey Administration

We administered the survey to students embarking on their clinical clerkships at our hospital (n=51) before the local session on digital professionalism; students had already attended the central session as described above. We surveyed students again at the end of their clerkship in April 2013. Data were maintained anonymously and without linking to protect student identity and facilitate truthful reporting.

#### Data Analysis

Responses were dichotomized to unprofessional (1,2) or professional (3,4,5), with neutral being considered professional for the purposes of analysis. Fisher test was used to determine significance of differences between pre- and post-clerkship surveys. All data were analyzed using SAS 9.3 (SAS Institute, Cary, NC). The primary outcome was participants’ change in opinions. Secondary outcomes included the observation of, and participation in, technology-related unprofessional behaviors.

In addition, for a subset of unprofessional behaviors where prevalence of participation was ≥30% (ie, medical record lookup outside of care and explicit instruction, use of third-party services with patient data, taking images of physical findings, conducting Web searches on patients), we analyzed post surveys to determine whether there was any correlation between observing behaviors and participating in behaviors. Where possible, we calculated relative risks (RR) and 95% CI.

We conducted a sensitivity analysis to determine how missing surveys would affect our results as not all students submitted end-of-the-year surveys, and we had avoided asking for linking information to preserve anonymity. This conservative analysis imputed answers for participants who did not return a post-clerkship survey, or who skipped a particular question, and biased responses toward the null.

## Results

### Survey Response

The response rates for the pre- and post-clerkship surveys were 96% (49/51) and 86% (42/49), respectively. Changes in perceptions regarding each behavior (pre- vs post-clerkship survey responses) and the post-clerkship observation and participation in each behavior are shown in Table 1.

### Privacy

When asked post-clerkship whether a medical student should access the record of a patient not under his or her care without explicit instruction to do so, respondents were less likely to consider the behavior as unprofessional (98-71%, *P*<.001). In total, 46% of students reported observing others perform this behavior and 32% participated themselves. All students who participated in the behavior had observed the behavior.

### Information Security

Fewer students perceived the use of third-party services (eg, Dropbox, Google Drive) for patient data to be unprofessional at the end of the year, reaching borderline statistical significance (94-80%, *P*=.06). The majority (58%) of students reported observing others use third party services (ie, online services not approved by the hospital and outside of the hospital firewall) and one-third reported doing so themselves (34%). All students who used these services observed the behavior in others. Fewer students considered the omission of passcode protection on personal devices as unprofessional (96-79%, *P*=.02), 26% observed others not doing so, and 8% did not do so themselves. Students who observed others not passcode protecting their devices were more likely to omit this protection as well, but this did not reach statistical significance. (RR=5.68, 95% CI 0.57-55.26).

### Communications

By the end of their clinical clerkship year, students were less likely to consider ignoring pages from nurses to be unprofessional (100-82%, *P*=.002). Fifty percent of students reported observing this behavior, though none reported participating in the behavior. We did not see any significant differences among students before and after the clerkship in regard to ignoring emails or pages from colleagues, with most considering such behaviors to be unprofessional. However, those who participated in these behaviors were more likely to have observed others participating in them (RR=16, 95% CI 2.25-113.59). All students who answered phone calls or looked at mobile devices in patient rooms or on rounds had observed the behaviors in others.

Other behaviors that students considered significantly less unprofessional after their clerkship year included conducting Web searches on patients (“Googling” patients, 57-29%, *P*=.01), “friending” patients on online social networks (100-90%, *P*=.04), and using a mobile device for non–work-related matters while in a patient’s company (100-90%, *P*=.04). Students who observed others “Googling” patients were more likely to participate in the behavior themselves (RR=3.65; 95% CI 1.29-10.32). Students who “Googled” residents or attendings (faculty physicians) were more likely to have observed the behavior (RR=2.65, 95% CI 1.15-6.10; RR=2.27, 95% CI 0.96-5.34, respectively). All students who “friended” attendings had observed the behavior, and students who “friended” residents tended to have observed the behavior (RR=3.48, 95% CI 0.52-23.30). All students who reported using Facebook at work or watching non–work-related videos at the hospital had observed others doing the same.

### Online Tone

Significantly fewer students considered the following types of online posts to be unprofessional: negative comments about patients (100-89%, *P*=.03), derogatory comments about nurses or hospital staff (100-89%, *P*=.03), and derogatory comments about residents or attendings (100-86%, *P*=.01). Students observed negative online comments about patients, residents, attendings, and nurses (prevalence ranging from 33- 42%), but denied participating in these behaviors (0%). Some students who observed inappropriate online behaviors from their colleagues, nurses, housestaff, and attendings (range=11.4-19%) did not give feedback about these behaviors. All students who observed others not giving feedback about inappropriate online behaviors reported not giving feedback themselves.

### Sensitivity Analysis

Our conservative sensitivity analysis resulted in the loss of statistical significance for many results where we observed a change in perceptions over the course of a clinical year. Notable exceptions included looking up medical records of patients outside of ongoing patient care or formal educational context (98-71%, *P*<.01), and not returning a page from a nurse (100-86%, *P*=.01).

**Table 1:** Perceptions of professionalism of technology-related behaviors pre- and post-clerkship.

Behavior		Preclerkship			Postclerkship				Observation and participation		
		n Responded	n (%) Unprofessional		n Responded	n (%) Unprofessional	*P* value		n (%) Observed	n (%) Participated
**Privacy**									
	Looking up the medical record of a patient who is not under your care without explicit instruction to do so	49	48 (98.0)		38	23 (60.5)	<.001		19 (46.3)	13 (31.7)
	Taking a photo or video of a patient’s physical findings	49	19 (38.8)		39	17 (43.6)	.67		34 (87.2)	13 (33.3)
	Sharing a photo or video of a patient’s physical findings	49	36 (73.5)		38	28 (73.7)	>.99		28 (71.8)	7 (17.9)
**Security**									
	Saving work that contains patient data to a 3rd party service	49	46 (93.9)		39	31 (79.5)	.06		22 (57.9)	13 (34.2)
	Not passcode protecting a personal device used for work	49	47 (95.9)		38	30 (78.9)	.02		10 (26.3)	3 (7.9)
	Downloading non-work related programs onto a work computer	49	38 (77.6)		37	25 (67.6)	.33		7 (18.9)	4 (10.8)
	Using a personal email address for professional communication	49	36 (73.5)		38	21 (55.3)	.11		22 (59.5)	15 (40.5)
	Using a professional email address for personal communication	49	13 (26.5)		37	10 (27.0)	>.99		30 (83.3)	27 (75.0)
**Communications**									
	Playing online games at work	48	45 (93.8)		40	31 (77.5)	.03		17 (43.6)	2 (5.1)
	Not returning a page from a nurse	49	49 (100.0)		38	31 (81.6)	.002		19 (50.0)	0 (0.0)
	Not returning a page from a colleague	49	49 (100.0)		37	34 (91.9)	.08		14 (36.8)	1 (2.6)
	Not returning a phone call or page from a patient	49	48 (98.0)		36	34 (94.4)	.57		12 (31.6)	2 (5.3)
	Not replying to an email requesting a response	49	48 (98.0)		39	34 (87.2)	.08		19 (50.0)	13 (35.1)
	Not replying to an email from a professor that requests a response	49	45 (91.8)		37	34 (91.9)	>.99		9 (25.0)	6 (16.7)
	Not replying to an email from a class administrator that requests a response	49	48 (98.0)		37	34 (91.9)	.31		12 (33.3)	9 (25.0)
**Social media and professional boundaries**									
	Answering a mobile phone while in a patient’s room	49	45 (91.8)		39	34 (87.2)	.50		31 (79.5)	2 (5.1)
	Using a mobile device for non-work related matters while on rounds	49	46 (93.9)		40	35 (87.5)	.46		28 (71.8)	8 (20.5)
	Using a mobile device for non-work related matters while in a patient’s company	49	49 (100.0)		40	36 (90.0)	.04		17 (43.6)	1 (2.6)
	Using Facebook at work	49	42 (85.7)		40	31 (77.5)	.41		35 (89.7)	15 (38.5)
	Watching non-work related videos at work	49	39 (79.6)		40	28 (70.0)	.33		34 (87.2)	16 (41.0)
	“Friending” a patient online	49	49 (100.0)		42	38 (90.5)	.04		2 (4.9)	0 (0.0)
	Accepting an “online friend request” from a patient	48	44 (91.7)		42	36 (85.7)	.51		2 (4.9)	0 (0.0)
	“Googling” a patient	49	28 (57.1)		42	12 (28.6)	.01		26 (63.4)	22 (53.7)
	“Friending” a resident online	49	5 (10.2)		39	7 (17.9)	.36		31 (77.5)	13 (32.5)
	“Googling” a resident	48	7 (14.6)		40	6 (15.0)	>.99		28 (68.3)	26 (65.0)
	“Friending” an attending online	49	24 (49.0)		41	18 (43.9)	.68		9 (22.0)	4 (9.8)
	“Googling” a physician	49	0 (0.0)		42	5 (11.9)	.02		34 (82.9)	36 (87.8)
**Online Tone**									
	Making negative comments about patients in online posts	49	49 (100.0)		37	33 (89.2)	.03		12 (33.3)	0 (0.0)
	Making derogatory comments about nurses or hospital staff in online posts	49	49 (100.0)		37	33 (89.2)	.03		15 (41.7)	0 (0.0)
	Making derogatory comments about residents or attendings in online posts	49	49 (100.0)		36	31 (86.1)	.01		13 (36.1)	0 (0.0)
	Making derogatory comments about peers in online posts	49	48 (98.0)		37	33 (89.2)	.16		15 (41.7)	0 (0.0)
**Accountability**									
	Not giving feedback to other students about inappropriate online behavior	49	36 (73.5)		36	21 (58.3)	.17		13 (36.1)	6 (16.7)
	Not giving feedback to residents about inappropriate online behavior	49	31 (63.3)		36	17 (47.2)	.19		10 (27.8)	7 (19.4)
	Not giving feedback to faculty about inappropriate online behavior	49	28 (57.1)		36	19 (52.8)	.83		9 (25.0)	4 (11.1)
	Not giving feedback to nurses about inappropriate online behavior	49	29 (59.2)		36	17 (47.2)	.38		11 (30.6)	6 (16.7)

## Discussion

### Principal Findings

Clinical clerkships are a critical time in the formation of medical students’ professional identities. Students constantly compare what they have been taught with what they see, and the influence of this “hidden curriculum” of medical school is thought to play an important role in the acculturation of students into the profession [13]. Our study, conducted after formal, interactive didactic sessions on the topic of digital professionalism, shows that students’ definitions of unprofessional behaviors change over the course of their clinical clerkships. Furthermore, observation of unprofessional activities is correlated with participation in these behaviors. Our findings are consistent with prior studies that looked at professional and ethical development of medical students and also showed changing perceptions of and participation in unprofessional behaviors after clinical clerkships [14-16,20]. To our knowledge, however, ours is the first study assessing medical students’ professional development in regard to digital professionalism.

### Legal and Regulatory Risk

Students encounter new risks for unprofessional and unethical behaviors with the use of electronic medical records and social media; these risks are rarely discussed or taught explicitly. Unfortunately, several of the behaviors we assessed have critical legal and regulatory implications, exposing trainees and medical centers to substantial ethical, legal, and financial risk. Snooping in charts, for instance, violates the privacy rule of HIPAA (Health Insurance Portability and Accountability Act of 1996) and subjects the individual and institution to fines, along with a loss of patient trust; students may be dismissed from school at the first occurrence of such a violation. Allowing data to “leak” from secure environments onto third-party cloud servers violates the Security Rule of HIPAA; third-party services should not be used, unless they are sanctioned by the organization, encrypted, and have signed business associate agreements ensuring compliance with regulations.

Given that policy tends to lag behind technology, educators and hospital leadership need to proactively assess and monitor behaviors of their students, and assess risk and compliance with expectations and existing regulations. Medical educators must understand the basis for unprofessional behaviors and provide education, support, and resources to make it easy for medical students to act professionally and ethically, even in these pressured environments. Organizations are beginning to create agreements with cloud-based providers that do ensure the security and auditability of protected health information while meeting the needs of users. This development is encouraging that health care entities are making it easy for users, including students, to do the right thing all the time.

### Ethical Considerations

Patients trust that medical providers, including students, will safeguard their information, upholding a central tenet of medical professionalism. Students also have an obligation to their patients and society to use their time in medical school most effectively to become competent clinicians. This sets up an ethical dilemma where a student feels that the use of a new but unsanctioned technology, like a third-party cloud service for preparing case presentations, outweighs the low but not negligible risk of a data breach. The law is clear in this case that data must be secured; in some cases, however, the law and policy are less clear.

Should students be allowed to look up the medical record of a patient not under their care for educational purposes? Electronic health records allow students the opportunity to see and follow different cases that they may not encounter on their own during medical school. Students may also follow patients longitudinally with the electronic health record, after their formal role in the patient’s care has ended, to learn the outcome [21]. However, it is unlikely that all patients would consider their records to be open to all to view, even when coming to teaching hospitals. Others have written on this potential ethical dilemma [22-24]. Furthermore, it appears from other data that some students are accessing their previous patients’ records for curiosity rather than more educationally related reasons. Our data show that, after their clerkship year, significantly fewer students felt it was unprofessional to look up medical records for patients not under their care, despite a nearly universal perception at the beginning; this suggests acculturation or normalization of the behavior occurs. Organizations and educational leaders should proactively discuss these dilemmas with their learners.

### Teaching and Modeling Digital Professionalism

Recognizing digital professionalism as an important component of medical education will allow integration into the classroom, the clinic, and simulation-based training, with competencies that are tested throughout medical training. However, integration of digital professionalism training must be done in a manner that truly instills students with the tangible tools and resources they need to act professionally. Professionalism training for students, faculty, and staff must shift from abstract descriptions such as “keep data private” to behaviorally oriented definitions, such as “encrypt mobile devices.” These definitions can be taught and refined as technology continues to evolve.

Although the introduction of a formal curriculum similar to the one we offer in our study may be a first step, our findings suggest that isolated sessions on professionalism are not sufficient to sustain perceptions and behaviors of professionalism [25]. Although we did not measure satisfaction with our sessions, students have generally considered these “on-doctoring” courses to be a frustrating, low-priority aspect of their training [26]. Furthermore, one-time educational sessions or written policies are not likely to sustainably promote professionalism. When Dawkins et al surveyed pediatric residents nationwide, the team found that residents viewed inappropriate social media postings not infrequently; more than half of the surveyed residents were unaware of social media policies despite nearly four-fifths of respondents having had some formal education around social media [27]. Taking a systems-level approach that goes beyond didactics and allows for professionalism training to be integrated more fully with clinical training may proactively promote proper behavior [17].

### Changing the “Hidden Curriculum”

A critical step in improving students’ performance and professional development is for faculty and staff to take a closer look at their own behaviors and expectations. Whereas the ultimate responsibility for unprofessional actions lies with the students themselves, faculty must hold themselves to a high standard. The more complex a setting and task, the bigger the discrepancy between what is explicitly taught in formal curricula and what is learned by students [26]. Until the culture of the hospitals and teams within which students function is changed, students will continue to receive conflicting messages on what is “ethical” and “professional.” Acting in a professional manner in the digital age requires a constant reflection and assessment of one’s tone and language and an active willingness to avoid electronic “shortcuts” that circumvent security. We found that students were more likely to engage in several unprofessional behaviors when they witnessed others doing so, emphasizing the need for deliberate change on the part of educators and entire hospital-based teams. Given the ease of taking such “shortcuts,” the lack of immediate repercussions when unprofessional actions are taken digitally, and the prevalence of unprofessional behaviors on social media across groups and professions, a “do as I say, not as I do” approach is unlikely to inculcate students with the necessary tools for success.

### Limitations

We conducted our study at a single site with a limited number of students. Our results may not be generalizable to other settings. We administered the survey anonymously to promote honest responses. However, this design limited our ability to perform paired analysis of pre- and post-clerkship surveys that might detect subtle differences. We performed a sensitivity analysis to conservatively account for missing responses. We did not formally pretest these questions with students and could not exclude differences in interpretation for some questions. However, questions from pre and posttest were kept identical with the same population, making it less likely to influence responses.

### Conclusions

Although technology is a useful tool to enhance teaching and learning, the ethical dilemmas and legal ramifications of its potential misuse require enhanced attention to learners’ beliefs and behaviors. True alteration of trainee behavior will require a cultural shift that includes continual education, better role models, and frequent reminders for faculty, house staff, students, and staff. Future studies should assess and compare various educational strategies for promoting professionalism.

## Appendix

Multimedia Appendix 1 Survey questions administered at the beginning and end of the clinical clerkship year.

Multimedia Appendix 2 Curriculum delivered during educational sessions at the beginning of the clinical clerkship year.

## Abbreviations

ePHI: electronic protected health information
