# Supplementary material for: Erosion of Digital Professionalism During Medical Students’ Core Clinical Clerkships
Source: JMIR Med Educ. 2017 May 3;3(1):e9. doi: 10.2196/mededu.6879 (PMC5438450; doi:10.2196/mededu.6879)
Supplement: Multimedia Appendix 1 [file mededu_v3i1e9_app1.pdf]

Appendix 1

Survey Questions

The students were asked in a pre and post test manner three questions for each of the following:

1. Have/Did you observe(d) the following behavior?
2. Have/Did you participate(d) in the following behavior?
3. Do you consider the behavior unprofessional?

Patients and Family Members:

1. Privacy
    1. "Friending" a patient online
    2. Accepting an "online friend request" from a patient
    3. "Googling" a patient
    4. "Friending" a resident online
    5. "Googling" a resident
    6. "Friending" an attending online
    7. "Googling" a physician
    8. Looking up the medical record of a patient who is not under your care without explicit instruction to do so
    9. Taking a photo or video of a patient's physical findings
    10. Sharing a photo or video of a patient's physical findings

b. Boundary Violations
    1. Answering a mobile phone while in a patient's room
    2. Looking up information on a mobile device in a patient's company
    3. Looking up information on a phone while on rounds
    4. Using a mobile device for non-work related matters while on rounds
    5. Using a mobile device for non-work related matters while in a patient's company
    6. Using Facebook at work
    7. Watching non-work related videos at work
    8. Playing online games at work

b. Communications:
    1. Not returning a page from a nurse
    2. Not returning a page from a colleague
    3. Not returning a phone call or page from a patient
    4. Not replying to an email requesting a response

b. Security:
    1. Saving work that contains patient data to a 3rd party service (e.g. Google Docs, Dropbox)
    2. Not passcode protecting a personal device used for work
    3. Downloading non-work related programs onto a work computer
    4. Using a personal email address for professional communication
    5. Using a professional email address for personal communication

b. Tone:
    1. Making negative comments about patients in online posts
    2. Making derogatory comments about nurses or hospital staff in online posts
    3. Making derogatory comments about residents or attendings in online posts
    4. Making derogatory comments about peers in online posts
b. Accountability:
    1. Not giving feedback to other students about inappropriate online behavior
    2. Not giving feedback to residents about inappropriate online behavior
    3. Not giving feedback to faculty about inappropriate online behavior
    4. Not giving feedback to nurses about inappropriate online behavior
