# Supplementary material for: Erosion of Digital Professionalism During Medical Students’ Core Clinical Clerkships
Source: JMIR Med Educ. 2017 May 3;3(1):e9. doi: 10.2196/mededu.6879 (PMC5438450; doi:10.2196/mededu.6879)
Supplement: Multimedia Appendix 2 [file mededu_v3i1e9_app2.pdf]

Educational Sessions.

Session 1: Central School-wide 45min session, held in auditorium

Learning Objectives	Learners will<br>1. Identify professional boundaries with respect to new technology, and ethical and professional consequences for transgressions<br>a. Pertaining to smart phones on wards<br>b. Pertaining to social media<br>2. Be able to describe cases where patients identified from internet searches where their personal cases were being discussed on social media platforms<br>3. Describe key steps for information security on the wards, regardless of specific hospital and policies<br>a. Encryption of smart phones<br>b. Avoidance of cloud-based personal information systems for electronic Protected Health Information
Format	• Overview of importance / didactic<br>• Case presentations / discussion / group reflection<br>• Review of general principles
Cases	Attached below

Cases For Discussion:

Case #1 (N.B. cases extracted from larger sample of cases created for a digital professionalism course. Students were exposed to these three cases during their orientation. Students were not subsequently re-exposed this academic year to the case series.)

Eduardo, a third-year HMS student, is at the midpoint of his surgical clerkship. He is considering a career as a surgeon and is quite nervous about performing well during daily case-based discussions with chief residents, which he considers to be "high stakes" opportunities to demonstrate his knowledge and interest. During these rounds, as soon as a symptom or a disease entity is named, Eduardo quickly starts looking up pertinent information on a new iPhone app that he finds very helpful. He always wants to be called on so that he can relate these newly identified facts, but no one ever calls on him. Other students are peppered with questions, but he is not. Eduardo is a relatively quiet student and does not volunteer information or question why he isn't being asked to participate in rounds. At his mid-clerkship feedback session, the clerkship directors read him the feedback from the chiefs, which includes several comments such as, "This student always seems uninterested in the cases we present. He is always texting friends, doing something like Facebook, or playing games on his smartphone. We have no idea if he is bored, distracted or simply not interested in surgery." Eduardo is devastated by this feedback and wants to improve.

Discussion:

a) What recommendations would you make for this student in terms of using the smartphone as a resource for rounds?
b) Could this misunderstanding have been avoided?
c) Can you articulate any potentially useful 'guidelines' that arose from your discussion?

Themes
1) Boundary violations with technology: being aware of when, where and how you use technology and what this might look like to others.
2) Generational differences in perception of technology between students and other clinicians. Importance of communication to clarify acceptable approaches to access information in real time educational and clinical settings.

Case #3

John, a third year medical student is learning how to stitch lacerations in the emergency room. One night, a young woman comes in with a cut on her hand after washing a glass with a chipped edge. An x-ray reveals no foreign body in the wound, and John is left alone with her to sew the wound. As he's putting in the sutures, the patient asks him if he has a girlfriend. John blushes, smiles, and says "most medical students don't have time for relationships" before quickly changing the subject. He finishes suturing while chatting about the Red Sox, and she subsequently goes home.

Two days later John gets a Facebook friend request from the patient with an attached message: "Hi John, do you remember me? Here's a picture of my hand… I wasn't sure if it should be this red. PS, want to catch a Sox game sometime?"

Discussion:
a) What should John do next?
b) What steps could John have taken to prevent this from happening?
c) Can you articulate any potentially useful 'guidelines' that arose from your discussion?

Themes:
1) Boundary violations with technology: Patient violating physician boundaries.
2) Implications of "Friending" or emailing a patient.

Case #4

Lauren, a 3rd year medical student, is excited about almost being done with her PCE year. Although she's still not sure what field she'd like to match in, Lauren is certain of one thing: she wants to focus on medical education as a central part of her career. On the advice of one of her mentors, Lauren purchased a camera that she always carries in her white coat to document interesting physical findings to use in future lectures. She's taken so many pictures that she has the process down to an art: she asks the patient's permission, takes the photos, and then takes a photo of the patient's wristband to make sure that she can look up the specifics of the patient's history later.

After taking the T to visit her friend in Cambridge, Lauren realizes that she left her white coat on the seat next to her. She calls the MBTA but they have no record of anyone returning a white coat or a camera.

Discussion:
a) What concerns does Lauren's lost camera raise?
b) What are the appropriate next steps?
c) What could she have done to avoid this situation?
d) Can you articulate any potentially useful 'guidelines' that arose from your discussion?

Themes:

1) Taking photos of patients or physical findings
2) Digital security of patient content
3) Taking responsibility for lost patient information

Session 2: Hospital Small Group Session

Learning Objectives	Learners will<br>1. Describe important local policies for encryption and information security<br>2. Describe best practices for storing ePHI for teaching cases
Format	• Interactive discussion, lasting 45 minutes. Starting with questions and ensuring that learning objectives met
